# *TP53*-Mutated Circulating Tumor DNA for Disease Monitoring in Lymphoma Patients after CAR T Cell Therapy

**DOI:** 10.3390/diagnostics11050844

**Published:** 2021-05-08

**Authors:** Liting Chen, Wei Mu, Jia Gu, Min Xiao, Liang Huang, Miao Zheng, Chunrui Li, Yi Xiao, Jianfeng Zhou, Xiaolu Long

**Affiliations:** Department of Hematology, Tongji Hospital, Tongji Medical College, Huazhong University of Science and Technology, Wuhan 430030, China; ltchen@tjh.tjmu.edu.cn (L.C.); muweicelltherapy@163.com (W.M.); meilixinqing1936@163.com (J.G.); xiaomin@tjh.tjmu.edu.cn (M.X.); lhuang@tjh.tjmu.edu.cn (L.H.); lyanlou@foxmail.com (M.Z.); cunrui5650@hust.edu.cn (C.L.); yixiao@tjh.tjmu.edu.cn (Y.X.); jfzhou@tjh.tjmu.edu.cn (J.Z.)

**Keywords:** non-Hodgkin’s lymphoma, chimeric antigen receptor T cell therapy, *TP53* mutation, circulating tumor DNA, droplet digital PCR

## Abstract

Chimeric antigen receptor T (CAR T) cell immunotherapy has shown remarkable efficacy in non-Hodgkin’s lymphoma (NHL) patients. Minimal residual disease (MRD) monitoring in NHL is essential after CAR T cell therapy, which can be achieved by monitoring circulating tumor DNA (ctDNA). The mutation of *TP53* in NHL has been suggested to be associated with a poor prognosis. However, whether *TP53*-mutated ctDNA can be used as a biomarker remains undetermined. In this study, a total of 40 patients with mutated *TP53* who received CAR T cell treatment were analyzed, and specific probes targeting 29 different *TP53* mutation sites in the 40 patients were designed and verified. Then, the presence of *TP53*-mutated ctDNA in longitudinal plasma samples was tracked by droplet digital PCR. Patients were stratified into two groups, favorable or unfavorable, based on their highest ctDNA level using a MAF cutoff of 3.15% according to the ROC curve. The unfavorable group had significantly worse PFS than the favorable group (*p* < 0.001). Our results suggest that patients with mutated *TP53* with a favorable ctDNA profile in the first trimester have better prognostic outcomes than patients with an unfavorable profile, and ctDNA can be a reliable predictor of the subsequent clinical outcome.

## 1. Introduction

Non-Hodgkin’s lymphoma (NHL) accounts for approximately 90% of all lymphomas and has a wide range of histological appearances and clinical features at presentation, making its diagnosis and treatment difficult [1]. Fortunately, in the era of immunotherapy, chimeric antigen receptor T (CAR T) cell therapy is an effective treatment for hematologic malignancies [2] and can overcome high-risk genomic lesions. Several CAR T cell clinical trials have shown remarkable efficacy in B cell non-Hodgkin’s lymphoma (B-NHL) [3]. To define the response to treatment and choose the best therapeutic strategy for NHL, minimal residual disease (MRD) is often regularly monitored. However, efficient methods for MRD monitoring that meet the standard of fast, inexpensive and sensitive disease detection are generally lacking.

Recently, circulating tumor DNA (ctDNA) detection has emerged as a promising minimally invasive technique to determine MRD and further assess the treatment response. Short-fragment DNA, derived from tumor cells, is a highly specific tumor marker [4]. During the processes of apoptosis, necrosis and secretion of NHL tumor cells, ctDNA is released into the bloodstream and can be measured by several methods. Droplet digital PCR (ddPCR) is a highly sensitive form of PCR based on water–oil emulsion droplet technology [5]. This technology can provide high precision and absolute quantification of nucleic acid target sequences and can be used for ctDNA monitoring [6]. Indeed, this method has been successfully utilized to validate mutations identified in ctDNA.

Previous studies have reported that the presence of *TP53* mutation is associated with a poor response to treatment, rapid disease progression and a decreased survival time in several cancers [7,8]. *TP53* mutation was also shown to be a promising prognostic factor in leukemia and lymphoma. For example, patients with *TP53* mutations have significantly shorter overall survival (OS) and progression-free survival (PFS) than patients without *TP53* mutations. Thus, *TP53* mutation monitoring could provide predictive information to guide targeted therapy for lymphoma patients [9,10,11]. However, there have been limited studies on whether *TP53*-mutated ctDNA in NHL can be measured by ddPCR and used as a suitable biomarker for MRD tracking and the further stratification of patients who receive CAR T cell therapy.

In the present study, we enrolled 370 NHL patients with *TP53* mutations confirmed by next-generation sequencing. *TP53*-mutated ctDNA was subsequently tracked by ddPCR in 40 patients who received CAR T cell therapy. We further investigated whether mutated *TP53* ctDNA is a suitable indicator for monitoring the response to treatment and the role of peak circulating *TP53* levels in predicting disease progression in these patients. We also analyzed the tumor diameter and other factors to explore their potential for predicting prognosis.

## 2. Materials and Methods

### 2.1. Patients

**Figure 1:**
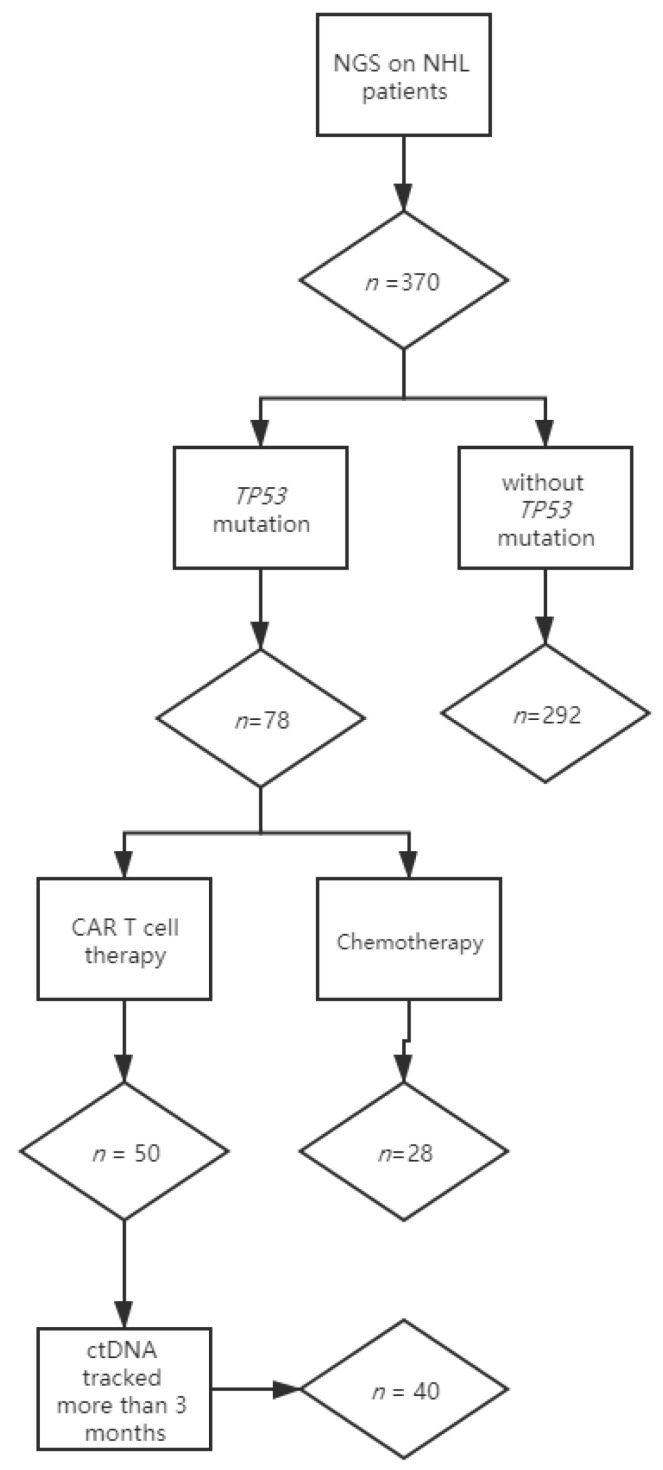
Flow chart describing the numbers of patients enrolled in the study. Lymphoma-focused next-generation sequencing was performed in 370 nonconsecutive lymphoma patients. *TP53* mutations were detected in 78 patients: 50 patients with mutated *TP53* received anti-CD19 and anti-CD22 CAR T cocktail therapy, and 28 patients received chemotherapy. Among the patients receiving CAR T cell treatment, 40 who had longitudinal plasma were included in this study, while 10 were excluded for insufficient ctDNA monitoring.

NHL patients with established *TP53* mutations who were treated with CAR T cell therapy and had ctDNA tracked at Tongji Hospital between July 2018 and December 2020 were included in this study (Figure 1). Appropriate informed consent was obtained from all patients under approved ethics committee protocols from Tongji Hospital (Protocols TJ-IRB20180813 and ChiCTR-OPN-16008526). All patients received a sequential infusion of an anti-CD19 and anti-CD22 CAR T cell cocktail (two single-specific, third-generation and two costimulatory domains from CD28, 4-1BB and the CD3ζ chain as the activation domain).

Patient demographics and clinicopathologic features, including the Ann Arbor stage, IPI score, tumor diameter, mutation sites, disease status and genetics, were collected (Supplementary Table S1). The investigator-determined objective response was assessed by the International Working Group (IWG) with the RECIL 2017 [12].

### 2.2. Collection and Processing of Peripheral Blood (PB)

Ten-milliliter peripheral blood (PB) samples were taken from NHL patients using EDTA K2 anticoagulation tubes after obtaining informed consent. After the sample was taken, the tube was inverted to mix the blood with EDTA in the collection tube to prevent coagulation. PB plasma was separated by centrifugation at 1600× *g* for 5 min within 24 h. The supernatant was collected by an additional centrifugation step at 1600× *g* for 10 min to remove cellular debris and then stored at −80 °C.

### 2.3. Cell-Free DNA Extraction

cfDNA was extracted from frozen plasma samples with the QIAamp Circulating Nucleic Acid Kit (Qiagen), and carrier RNA was added before lysis. A median sample volume of 4.8 mL (range 3.5–7.0 mL) of plasma was used for cfDNA extraction. Control circulating DNA was extracted from a pool of plasma from several healthy individuals. After extraction, cfDNA was quantified using a Qubit fluorometer 3.0 and a highly sensitive DNA detection kit (Invitrogen, Life Technologies, Carlsbad, CA, USA). The extracted cfDNA was stored at −20 °C until ddPCR.

### 2.4. Probe Design

Patient-specific TaqMan MGB (Primer Express 3.0.1) assays were designed for mutated and wild-type *TP53* sequences and labeled with FAM or VIC fluorophores separately (Applied Biosystems, Foster City, CA, USA). To avoid *TP53* mutation sites residing in the WT *TP53* probe, we chose the 5′-UTR as the WT probe-targeting region. The primer and probe sequences used in the reference assays were as follows: forward primer, 5′-CCCTCCCATGTGCTCAAGAC-3′; reverse primer, 5′-CTGGACGGTGGCTCTAGACTTT-3′; and probe, 5′-VIC-CTAAAAGTTTTGAGCTTCTC-3′. The sequences of the mutated primers and probes are shown in Table 1.

### 2.5. Droplet Digital PCR

ddPCR analysis was performed in a total reaction volume of 20 μL: 10 μL of ddPCR™ Supermix (without dUTPs; Bio-Rad, Hercules, CA, USA), 1 μL of forward primers (10 μmol/L), 1 μL of reverse primers (10 μmol/L), 2 μL of probes (2.5 μmol/L) and 2 uL of ctDNA (20 ng/uL). The sample was separated into approximately 20,000 compartments within oil droplets and then amplified on an ABI Thermal Cycler according to the following protocol: 5 min at 95 °C, followed by 40 cycles of 30 s at 95 °C and 1 min at 60 °C, followed by an enzyme deactivation step for 10 min at 98 °C. Following PCR, the PCR plate was transferred to a droplet reader, allowing specific fluorescence detection (VIC for the wild-type and FAM for the mutant), and analyzed with QuantaSoft version 1.7.4 (Bio-Rad Laboratories, Hercules, CA, USA).

### 2.6. QX200 Droplet Reader

QuantaSoft™ analysis software version 1.7.4 (Bio-Rad Laboratories GmbH, Munich, Germany) enables the detection of the mutant copy number and mutant allele fractions in samples. In the analysis, a threshold between positive and negative droplet clusters (both WT and mutant) was manually set, and the threshold was used to determine positive amplification. Poisson correction was used.

The level of mutated *TP53* ctDNA was quantified as the *TP53* mutant allele fraction (*TP53* MAF), defined as the *TP53* MAC divided by the *TP53* TAC. Total circulating cell-free DNA was measured as the *TP53* total allele count (*TP53* TAC), defined as the sum of the mutant and wild-type copies of amplified *TP53*. The number of mutated *TP53* amplifiable copies (*TP53* mutant allele count (*TP53* MAC)), defined as the number of single-stranded fragments of DNA amplified containing the mutation of interest, was determined.

The sequential ctDNA samples were quantitatively tracked. Positive and negative controls were included in each run. Serum samples were run in duplicate to screen additional DNA.

### 2.7. Statistics

The characteristics of patients in different ctDNA subgroups were compared using the χ2 test and the Spearman rank correlation. Frequencies and percentages (by group) along with their corresponding *p* values are reported. The optimum cutoff points for determining disease progression were identified using receiver operating characteristic (ROC) curves, and the area under the curve (AUC) was calculated to assess the performance of the *TP53* MAF in disease progression. The *Youden* index (sensitivity + specificity − 1) was used to identify the optimal cutoff values, and the *TP53* MAF value corresponding to the maximal Youden index was then designated as the cutoff. PFS was calculated from the time of diagnosis to the time of progression or death from any cause. PFS curves of different groups were analyzed using the Kaplan–Meier method, and differences were compared using the log-rank (Mantel-Cox) test. The hazard ratio (HR) and 95% CI of the ctDNA profile and tumor diameter were calculated using Cox regression analysis. In all analyses, two-sided tests were used, and a *p* value less than 0.05 was considered statistically significant. Data were analyzed using IBM SPSS Statistics for Windows version 25.0 (IBM Corp, Armonk, NY, USA) and GraphPad Prism version 7.0 (GraphPad Software Inc., San Diego, CA, USA).

## 3. Results

### 3.1. Monitoring Strategy

Lymphoma-focused next-generation sequencing was performed on tumor biopsies at diagnosis in 370 nonconsecutive lymphoma patients. *TP53* mutations were detected in 21% (78/370) of patients, consistent with previous studies [13]. Fifty patients with mutated *TP53* received anti-CD19 and anti-CD22 CAR T cocktail therapy, and 28 patients received chemotherapy. Among the patients receiving CAR T cell treatment, 40 who had longitudinal plasma were included in this study, while 10 were excluded for insufficient ctDNA monitoring (less than 3 months or frequency of fewer than 3 times) (Figure 1).

The baseline characteristics of the 40 traced patients are summarized in Table 2. The median age was 43.5 years (range 27–64), with 95% of patients being ≤ 60 years old. All patients manifested with an aggressive clinical course: 19 patients had primary refractory disease, 21 patients relapsed at least once and the chromosome 17p deletion or *IgH*/*MYC* translocation was detected in 17 patients (42.5%). In terms of tumor diameter, 18 patients had a tumor diameter larger than 5 cm, and the tumor diameters of the remaining 22 patients were smaller than 5 cm. Regarding prior therapies, all 50 patients underwent at least one cycle of chemotherapy before CAR T cell treatment, and the objective response rate to CAR T cell therapy was 65% (25/40).

A total of 219 plasma samples were collected from 40 traced patients, and their median and mean follow-up times were 3 and 6.35 months, respectively. Longitudinal samples were collected over several time points from treatment commencement. Among these 40 patients, 17 were followed up for at least 6 months, and 8 were followed for at least 12 months.

### 3.2. Distribution of TP53 Mutations in the Patient Cohort

Among the 40 patients, 36 carried a single nucleotide missense mutation, patient 6 carried a splicing mutation, patient 13 carried a nucleotide insertion and two (patient 22 and patient 28) carried nucleotide deletions. Almost all the missense mutations were predicted to be deleterious to the structure and function of the *TP53* protein based on the PROVEAN score (Supplementary Table S2). Protein Variation Effect Analyzer (PROVEAN) is a software tool which predicts whether an amino acid substitution or indel has an impact on the biological function of a protein [14]. It has been reported that *TP53* mutation in the DNA-binding domain (codons 94–292) is predictive of poor survival in NHL patients [15], so the distribution of *TP53* mutation sites in 40 patients was analyzed. In this study, 25 of the 40 (62.5%) mutations resided within the DNA-binding domain (codons 94–292). These included 13 mutations in loop-L3 (codons 237–250) that interacts with the DNA minor groove, 10 mutations in the LSH helix motif (codons 119–135 and 272–287), which interacts with the DNA major groove and 2 mutations in loop L2, which enhances the binding affinity of *TP53* to the DNA helix. *TP53* mutation sites in 23 patients were located in loop-L3 or the loop-sheet-helix, which are associated with a poor prognosis. Moreover, 17 patients harbored hotspot mutations (Arg175, Gly245 Arg248, Arg273 and Arg282), which have been previously described as recurrent in non-Hodgkin’s lymphoma and other cancers [16].

### 3.3. Validation of ddPCR Assays

A total of 29 probes targeting *TP53* mutation sites were designed and validated. To test the performance of the assays (primers and probes), circulating DNA from 12 healthy volunteers was used as a negative control, and the false positive rate (defined as the average MAF falsely detected in the healthy samples) and threshold (determined as the upper 95% confidence interval of the assay-specific false positive rate) of each probe were calculated (Supplementary Table S2). The median false positive rate was 0.0001 (range from 0 to 0.0005, mean 0.0001). The thresholds of seven probes were 0, and the median threshold was 0.0002 (range 0 to 0.0015, mean 0.0003). These results indicate that the performance characteristics of these probes targeting mutated *TP53* sites are reliable.

### 3.4. ctDNA Monitoring Results (TP53 MAF) in NHL Patients

The sequential plasma MAFs of *TP53*-mutated ctDNA were quantitatively tracked by ddPCR. We analyzed the diagnostic efficiency by the ROC curve according to the highest *TP53* MAF in the first trimester after infusion, and disease progression was selected as a clinically significant endpoint. The ROC plot identified MAF = 3.15% (AUC = 0.96, *p* < 0.0001) as the most accurate threshold for predicting disease progression (Supplementary Figure S1).

**Figure 2:**
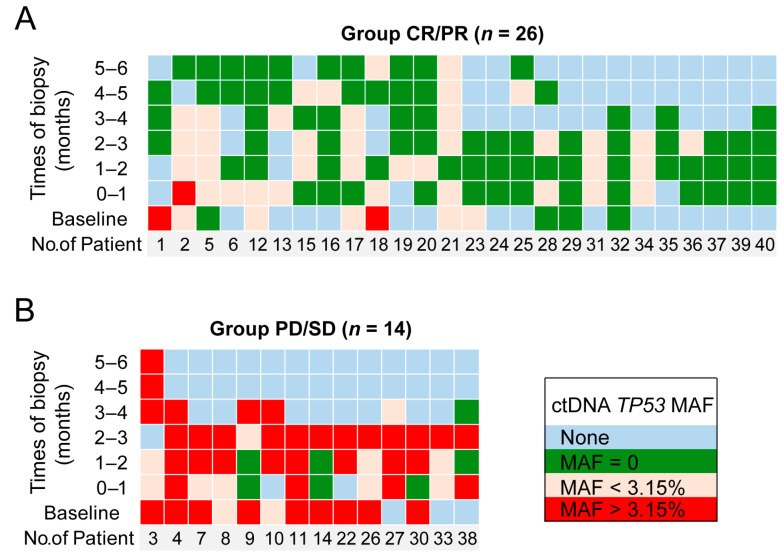
The *TP53* MAF predicts disease progression. Each column demonstrates the longitudinal ctDNA results of an individual patient for up to 6 months. The green squares represent undetectable ctDNA levels. The yellow and red squares represent *TP53* MAFs less than and greater than 3.15%, respectively. The blue squares represent no samples detected at these time points. (**A**) Group of patients who achieved complete or partial response; (**B**) group of patients with stable or progressive disease.

A total of 26 patients who achieved complete or partial response were defined as group CR/PR and are shown in Figure 2A. Among these patients, only one (No. 2) had an increased ctDNA level (*TP53* MAF > 3.15%) after therapy during the first month that later decreased. Among the other 25 patients, eight had low baseline ctDNA levels (*TP53* MAF < 3.15%) that remained low or converted to zero in the first trimester, and two had high baseline ctDNA levels (*TP53* MAF > 3.15%) that decreased (*TP53* MAF < 3.15%) following the first trimester. The remaining 15 patients had no plasma samples at baseline, but in the first trimester, their *TP53* MAFs were undetectable or less than 3.15%.

**Figure 3:**
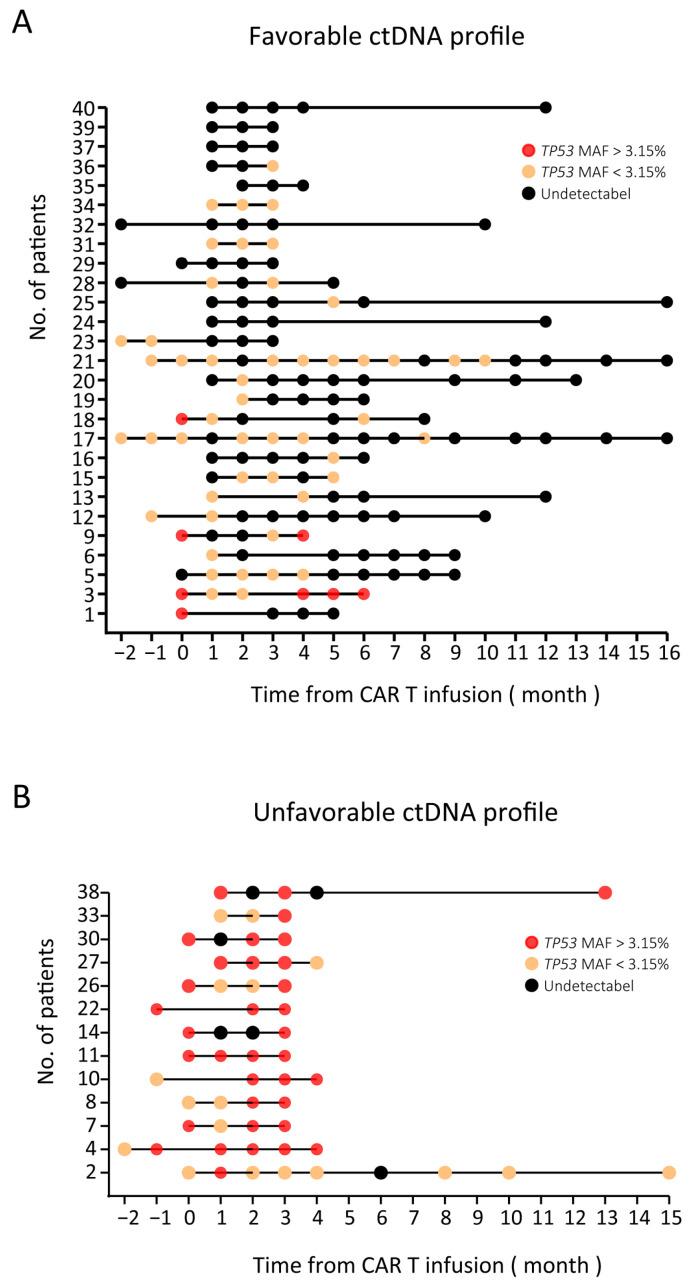
Overview of ctDNA results (*TP53* MAF) in 40 NHL patients. (**A**) A favorable ctDNA profile: undetectable ctDNA at baseline that remained undetectable or detectable ctDNA at baseline that became undetectable or decreased less than 3.15% during treatment. (**B**) An unfavorable ctDNA profile: detectable ctDNA at baseline that remained stable or increased during treatment. Black circles: undetectable ctDNA levels; yellow circles: *TP53* MAF less than 3.15%; red circles: *TP53* MAF greater than 3.15%.

Fourteen patients with stable or progressive disease were defined as group PD/SD and are shown in Figure 2B. In this group, nine patients had high baseline ctDNA levels (*TP53* MAF > 3.15%): those of seven patients remained high in the first trimester, and those of the other two patients (patient 3 and patient 9) decreased to less than 3.15% in the first trimester and rebounded in the fourth month. The ctDNA levels of the remaining five patients in group PD/SD all rose to a high level (greater than 3.15%) in the first trimester. These results showed that for CR/PR patients, their *TP53* MAF was mostly (25 of 26, 96.2%) undetectable or less than 3.15%. On the other hand, for PD/SD patients, their *TP53* MAF was mostly (12 of 14, 85.7%) greater than 3.15%. Then, patients were stratified into two groups depending on their highest ctDNA level in the first trimester after CAR T cell infusion using a MAF cutoff of 3.15%. Patients whose highest *TP53* MAF was less than 3.15% in the first trimester were considered to have a favorable ctDNA profile. Conversely, patients whose highest *TP53* MAF was greater than 3.15% in the first trimester were considered to have an unfavorable ctDNA profile (Figure 3). 

The objective response rate was significantly higher in the favorable group than in the unfavorable group (25/27 (92.6%) and 1/13 (7.7%), respectively, Supplementary Table S3). In the favorable group, 20 patients achieved CR, and 5 patients achieved partial response, while in the unfavorable group, only 1 patient achieved PR, and 12 patients showed disease progression.

### 3.5. Prognostic Value of TP53 in ctDNA for Patients Receiving CAR T Cell Therapy

To investigate whether the *TP53*-mutated ctDNA level in the first trimester after CAR T cell infusion can indicate prognosis, we compared two groups of ctDNA profiles to estimate PFS.

**Figure 4:**
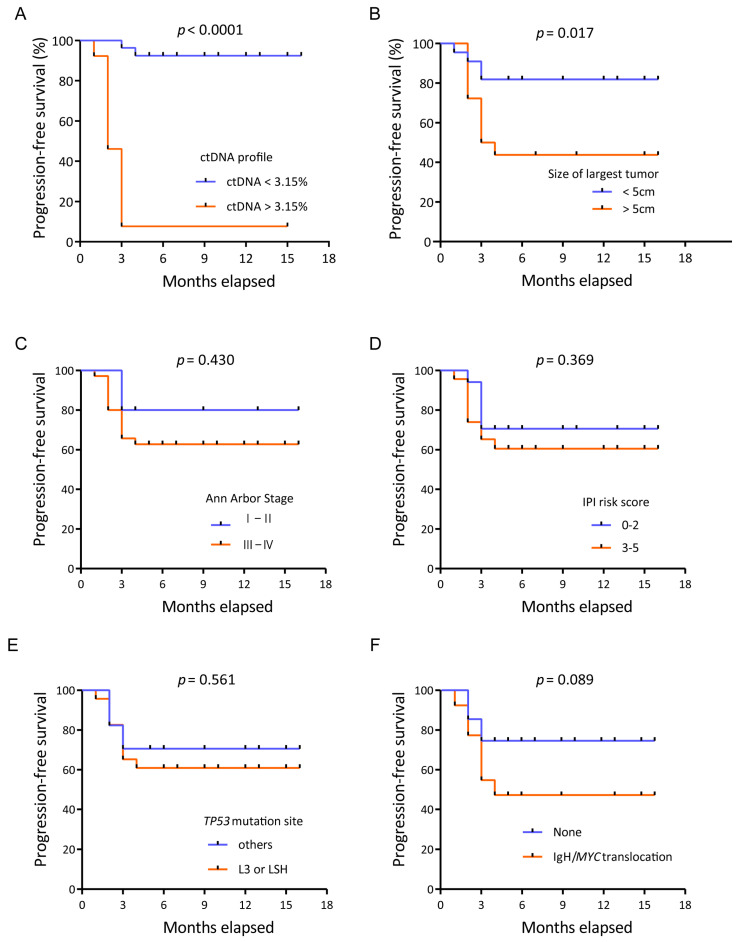
Kaplan–Meier plots of progression-free survival. (**A**) Kaplan–Meier plots of progression-free survival according to the ctDNA profile. Progression-free survival of patients with an unfavorable ctDNA profile versus those with a favorable ctDNA profile. (**B**) Kaplan–Meier plots of progression-free survival according to the tumor diameter. Progression-free survival of patients with a large tumor diameter versus those with a small tumor diameter. (**C**) Kaplan–Meier plots of progression-free survival according to the Ann Arbor stage. (**D**) Kaplan–Meier plots of progression-free survival according to the IPI risk score. (**E**) Kaplan–Meier plots of progression-free survival according to the *TP53* mutation sites. (**F**) Kaplan–Meier plots of progression-free survival according to the *IgH*/*MYC* translocation.

A significant hazard ratio (HR) was established for the ctDNA profile (Table 3). The risk of disease progression in patients with an unfavorable ctDNA profile was 19 times higher than that in patients with a favorable ctDNA profile (HR 19.45, *p* < 0.0001). Kaplan–Meier analysis showed that the unfavorable group also had a significantly worse PFS (median PFS, 3 months) than the favorable group (median PFS, not reached; *p* < 0.0001; Figure 4A). At the time of data cutoff, nine patients had disease progression: two patients in the favorable group and seven patients in the unfavorable group.

Other potential factors, such as the tumor diameter, Ann Arbor stage, IPI risk score, *TP53* mutation site and *IgH*/*MYC* translocation, were also analyzed. The largest tumor diameter was also found to be predictive, and a tumor size over 5 cm predicted poor PFS (hazard ratio 3.44, *p* = 0.017) according to the univariate analysis (Figure 4B). However, the Ann Arbor stage, IPI score, *TP53* mutation site and *IgH*/*MYC* translocation were not associated with progression-free survival (Figure 4C–F).

### 3.6. ctDNA Levels Can Reflect Tumor Burden

**Figure 5:**
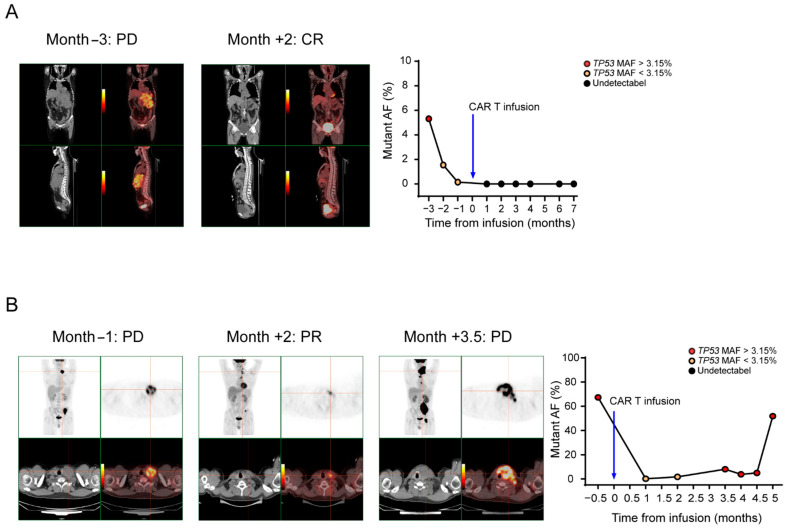
*TP53* ctDNA monitoring of the therapeutic response. Time courses of plasma molecular disease during therapy; red, positive molecular disease; black, negative molecular disease. (**A**) Patient 23 was diagnosed with DLBCL, and the disease burden declined as the patient achieved CR after CAR T cell therapy. Plasma molecular disease at baseline was positive, decreased to less than 3.15% and then dropped to zero. (**B**) Patient 3 was diagnosed with FL, and the disease burden declined in the first 2 months as the patient achieved PR after CAR T cell therapy. However, the patient relapsed as the disease burden rose in the third month. Plasma molecular disease at baseline was positive, decreased to less than 3.15% in the first 2 months and then increased to greater than 3.15% in the third month.

To study the ability of ctDNA to reflect tumor burden, we assessed the concordance of consecutive ctDNA levels with the radiology results. As shown in Figure 5A, patient 23 was diagnosed with DLBCL, and the disease burden declined as the patient achieved CR after CAR T cell therapy. Plasma molecular disease at baseline was positive, decreased to less than 3.15% and then dropped to zero. As shown in Figure 5B, patient 3 was diagnosed with FL, and the disease burden declined in the first 2 months as the patient achieved PR after CAR T cell therapy. However, the patient relapsed as the disease burden rose in the third month. Plasma molecular disease at baseline was positive, decreased to less than 3.15% in the first two months and then increased to greater than 3.15% in the third month. ctDNA levels change as the tumor burden changes, with escalating levels in PD, low levels in PR and declining or undetectable levels in CR. Moreover, the mean time to detect disease progression with the *TP53* MAF was shorter than that with CT imaging in 14 PD patients, specifically, there were four patients that had a positive result with ctDNA level earlier than that with CT imaging (Supplementary Figure S2), with a mean lead time of 1.3 months (range 1 to 2 months). This result suggests that occult disease can be detected by plasma ctDNA levels before detection by PET/CT. These results also show that ctDNA monitoring has better specificity for relapse detection than CT imaging.

## 4. Discussion

CAR T cell immunotherapy has been successfully used in the treatment of hematologic malignancies, demonstrating remarkable antitumor efficacy and producing tumor regression in the majority of treated patients [17,18,19]. There is a high need for the identification of reliable novel biomarkers that can predict the treatment response and probability for long-term PFS, detect early disease progression and assist in timely clinical decision making. Conventional monitoring methods for non-Hodgkin’s lymphoma (NHL) patients receiving CAR T cell therapy include bone marrow/tumor biopsy and PET/CT [20]. Traditional biopsies are invasive and may bring a risk of injury or complications to patients. Furthermore, tumor biopsies have spatial and temporal limitations that can provide only limited information. In addition, PET-CT always detects disease areas depending on the macroscopic tumor load, but microscopic disease may be missed; therefore, patients may be misjudged as achieving complete remission. In addition, PET/CT imaging may be expensive, and patients are exposed to radiation [21]. Traditional assessment methods have encountered limitations, and novel disease assessment techniques have emerged.

Several studies have shown the utility of ctDNA as a diagnostic, predictive and prognostic biomarker. It has been reported that the molecular response measured by deep targeted cfDNA sequencing after infusion is significantly associated with ongoing disease progression [22]. Quantification of the clonotypic VDJ rearrangement in ctDNA from DLBCL patients was also reported; according to this study, the clinical progression hazard ratio was significantly higher for patients with detectable ctDNA than for patients with undetectable ctDNA [23]. These studies concluded that ctDNA analysis reveals biological factors that underline the clinical outcomes of lymphoma patients and can facilitate individualized therapy. In our study, similar findings were obtained: for CR/PR patients, their ctDNA profiles were mostly (25 of 26, 96.2%) favorable. On the other hand, for PD/SD patients, their ctDNA profiles were mostly (12 of 14, 85.7%) unfavorable. A favorable ctDNA profile (during the first 0–3 months) correlated with prolonged PFS. Patients with an unfavorable ctDNA profile had a 19-fold increased risk of disease progression (hazard ratio 19.45, *p* < 0.0001). According to the HR, the ctDNA profile was a better predictor of PFS than other indicators, such as the tumor diameter, Ann Arbor stage, IPI risk score, *TP53* mutation site and *IgH*/*MYC* translocation (Table 3). These findings were also consistent with other studies that reported that the median time for ctDNA to predict cancer recurrence was several months earlier than that for CT imaging [21]. Plasma ctDNA levels and radiographic results always matched in our study. Moreover, among the patients who achieved PD, the mean time to detect disease progression with ctDNA was shorter than that with CT imaging, possibly because, compared with CT imaging or tissue biopsy, liquid biopsy can greatly shorten the examination interval. CT imaging provides a macroscopic estimate of the overall disease burden but is limited by its low sensitivity and tumor specificity, while ctDNA represents changes in the tumor genome at the molecular level that may occur earlier than macroscopic metastasis or progression [24]. In summary, ctDNA monitoring can increase the lead time and provide a crucial early opportunity for clinical intervention that can be vitally important in relapsed NHL patients who may still have the chance for a cure.

The methods of ctDNA analysis in this study include deep sequencing and ddPCR. We performed next-generation sequencing on lymphoma patients, *TP53* mutations were detected in 21% (78/370) of patients, and then we tracked the presence of ctDNA in plasma over time from longitudinal plasma by droplet digital PCR. The technology of NGS could be used to sequence multiple genes and variants simultaneously, and it also allows the identification of novel genetic modifications and analysis of clone evolution [25]. ddPCR can detect specific genetic changes, it has increased site-specific detection sensitivity and the experiments are easy to set up and do not require complex informatics support for analysis [26]. In addition, ddPCR has been successfully utilized to validate mutations identified by sequencing technologies in ctDNA. The combination of these two methods will give full play to the advantages of both, so as to benefit patients more.

*TP53* gene defects are common in almost all types of human cancer [27,28]. The P53 protein encoded by the *TP53* gene is an important tumor suppressor that mediates cell cycle arrest, DNA repair, apoptosis, aging and autophagy under cellular stress [15]. Nonsynonymous *TP53* mutations alter the P53 protein sequence and structure, disrupt its function and are the most common mechanism that inactivates *TP53* [29,30]. In this study, we tested 29 specific *TP53* mutations, most of which resided within the DNA-binding domain and have been reported to be associated with a poor prognosis in various cancers, including NHL [31]. Primers and MGB probes were designed for 29 different mutations. ctDNA from healthy volunteers was used as a control to verify the specificity of the probes. False positive rates were calculated, and thresholds were set to the upper 95% CI of the false positive rates. If the result was lower than the threshold, the patient was determined to be ctDNA negative. All 29 assays (primers and probes) were designed by Primer Express 3.0.1, and the lowest penalty was chosen. Our work confirms and validates the utility of these assays, and we also provide here the primer and probe sequences used to target of all of the 29 *TP53* mutations.

To date, the efficacy of ctDNA monitoring after CAR T cell therapy has been worth exploring. Here, we provide evidence that ctDNA can be used to monitor the response to CAR T cell therapy, detect MRD and predict prognosis. In this study, patients were stratified into two groups based on their highest ctDNA level in the first trimester after infusion using a MAF cutoff of 3.15% by the ROC plot (*p* < 0.0001). Patients in the unfavorable group had significantly worse PFS than patients in the favorable group (*p* < 0.001). ctDNA was superior to other baseline clinical parameters associated with the response and prognosis, including the Ann Arbor stage and IPI score, and even better than the tumor diameter. This study provides evidence that plasma ctDNA can be a reliable predictor of the subsequent clinical outcome, that it will help clinicians identify unfavorable patients after the initial treatment and that early intervention can be directed by ctDNA detection.

## 5. Conclusions

We showed the potential for the *TP53* mutated ctDNA to identify NHL patients with an expected poor or good response after CAR T cell therapy, and our results suggest that *TP53*-mutated ctDNA within the first trimester can predict disease progression and the PFS of NHL patients receiving CAR T cell therapy and that ctDNA levels can also reflect the change in tumor burden.

## Figures and Tables

**Table 1:** *TP53* mutation sites and ddPCR assay information.

No. of Patient	Protein Change	Nucleotide Change	Mutant Probe	Forward Primer	Reverse Primer
28	p.E336fs	c.1007_1020del	TGGGCGTGTTCCGAGAG	CCTCCTCTGTTGCTGCAGATC	TGAGTTCCAAGGCCTCATTCA
40	p.S106R	c.318C > G	CAGGGCAGGTACGGT	CTGTCCCTTCCCAGAAAACCT	GGCTGTCCCAGAATGCAAGA
6	-	c.376-1G > A	TTCCTACAATACTCCCCT	TGACTTTCAACTCTGTCTCCTTCCT	GCTGCACAGGGCAGGTCTT
3	p.Y126N	c.376T > A	CAGGACTCCCCTGCC	CAACTCTGTCTCCTTCCTCTTCCT	GGCCAGTTGGCAAAACATCTT
24	p.A138V	c.413C > T	CAGGTCTTGACCAGTT	ATGTGCTGTGACTGCTTGTAGATG	GCCCTCAACAAGATGTTTTGC
1 32	p.W146 *	c.437G > A	TGCAGCTGTAGGTTGAT	TCAACAAGATGTTTTGCCAACTG	ATGTGCTGTGACTGCTTGTAGATG
15	p.Y163C	c.488A > G	CCATCTGCAAGCAG	CTGCCCTCAACAAGATGTTTTG	CCTCACAACCTCCGTCATGTG
20 23	p.R175H	c.524G > A	ATGGGCCTCCAGTTC	TGGAGTCTTCCAGTGTGATGATG	CAACTACATGTGTAACAGTTCCTGCAT
19	p.D208Y	c.622G > T	ATGGGTTTAACTATTTCACAG	ACCCCATGAGATGTGCAAAGTA	GCCTCTGTAAGCTTCAGTTTTTTCA
25	p.R213 *	c.637C > T	CACTTTTTGACATAGTGTG	CGTGTGGAGTATTTGGATGACAGA	AGACCTCAGGCGGCTCATAG
26	p.R213Q	c.638G > A	CACTTTTCAACATAGTG	CGTGTGGAGTATTTGGATGACAGA	AGACCTCAGGCGGCTCATAG
5	p.I232N	c.695T > A	TACCACCAACCACTACA	TGGGCCTGTGTTATCTCCTAGGT	CCATGCAGGAACTGTTACACATG
30	p.Y234D	c.700T > G	ACCATCCACGACAAC	TCTCCTAGGTTGGCTCTGACTGT	CCATGCAGGAACTGTTACACATG
33	p.Y236D	c.706T > G	CACTACAACGACATGTGTAA	TTGGCTCTGACTGTACCACCAT	TGGGCCTCCGGTTCATG
9	p.C238W	c.714T > G	CATGTGGAACAGTTCC	TCTGACTGTACCACCATCCACTACA	TGGGCCTCCGGTTCATG
14 36	p.N239D	c.715A > G	TGTGTGACAGTTCC	TGACTGTACCACCATCCACTACAA	GATGGGCCTCCGGTTCAT
11	p.C242Y	c.725G > A	TGTAACAGTTCCTACATG	CTGTACCACCATCCACTACAACTACA	TGGGCCTCCGGTTCATG
7 13 31	p.G245S	c.733G > A	CCGGTTCATGCTGCC	TGGAGTCTTCCAGTGTGATGATG	AACTACATGTGTAACAGTTCCTGCAT
35	p.R248W	c.742C > T	ATGGGCCTCCAGTTC	TGGAGTCTTCCAGTGTGATGATG	CAACTACATGTGTAACAGTTCCTGCAT
8 18 21 27	p.R248Q	c.743G > A	ATGGGCCTCTGGTTCA	TGGAGTCTTCCAGTGTGATGATG	CAACTACATGTGTAACAGTTCCTGCAT
39	p.R249S	c.747G > C	AACCGGAGCCCCAT	GTACCACCATCCACTACAACTACATGT	GGCTCCTGACCTGGAGTCTTC
22	p.T253Pfs * 92	c.756delC	CCATCCTACCATCATC	GCATGGGCGGCATGAA	GGCTCCTGACCTGGAGTCTTC
37	p.E258D	c.774A > C	CACTGGACGACTCC	TTGGCTCTGACTGTACCACCAT	TGTGCAGGGTGGCAAGTG
10	p.R267W	c.799C > T	CTACTGGGATGGAACAG	GCTTCTCTTTTCCTATCCTGAGTAGTG	GCACAAACACGCACCTCAAA
17	p.R273C	c.817C > T	CTTTGAGGTGTGTGTTT	TGGTAATCTACTGGGACGGAACA	CTCTGTGCGCCGGTCTCT
2 4 34 38	p.R273H	c.818G > A	AGGTGCATGTTTGTGCC	TCTACTGGGACGGAACAGCTTT	CTCTGTGCGCCGGTCTCT
16	p.D281V	c.842A > T	TGGGAGAGTCCGGCG	AGCTTTGAGGTGCGTGTTTGT	TGCGGAGATTCTCTTCCTCTGT
29	p.R282W	c.844C > T	TGTGCGCCAGTCT	CCTTTCTTGCGGAGATTCTCTTC	AGCTTTGAGGTGCGTGTTTGT
12	p.R282-R283insHR	c.847-848insATCGGG	CCGGTTCATGCTGCC	TGGAGTCTTCCAGTGTGATGATG	AACTACATGTGTAACAGTTCCTGCAT

p.W146 *, p.R213 *, nonsense mutation; p.T253Pfs * 92, frameshift mutation.

**Table 2:** Patient and disease characteristics at baseline.

Characteristic	Favorable	Unfavorable	Total	*p* Value
**Age**, median (range)			43.5 (27–64)	
**Age group**, years				
Younger than 60 y	26	12	38 (95%)	0.59
At least 60 y	1	1	2 (5%)	
**Gender**				
Male	21	11	32 (80%)	0.61
Female	6	2	8 (20%)	
**IPI risk score**				
0–2	13	4	17 (42.5%)	0.30
3–5	14	9	23 (57.5%)	
**Ann Arbor stage**				
I–II	4	1	5 (12.5%)	0.95
III–IV	23	12	35 (87.5%)	
**Volume of disease**				
<5 cm	19	3	22 (55%)	0.01
>5 cm	8	10	18 (45%)	
**Disease status**				
Primary refractory	12	7	19 (47.5%)	
First relapse	8	4	12 (30%)	0.74
≥Second relapse	7	2	9 (22.5%)	
**Genetics**				
*IgH*/*MYC* translocation	7	3	13 (32.5%)	0.72
*TP53* mutation with del (17p)	5	2	7 (17.5%)	0.81
**All *TP53* mutations**				
Outside L3 and LSH	12	5	17 (42.5%)	0.72
Loop-L3 and LSH motifs	15	8	23 (57.5%)	

HSCT, hematopoietic stem cell transplantation; CAR T, chimeric antigen receptor T cell therapy; LSH, loop-sheet-helix. *p* value is presented to two decimal places or two significant digits.

**Table 3:** Univariate analysis of progression free survival.

Characteristic	Variable	Progression-Free Survival	
		Hazard Ratio (95% CI)	*p* Value
ctDNA	Unfavorable/favorable	19.45 (5.62–66.82)	<0.0001
Volume of disease	>5 cm/<5 cm	3.44 (1.19–9.95)	0.017
Ann Arbor stage	Ⅲ–Ⅳ/I–II	2.11 (0.47–9.57)	0.430
IPI risk score	3–5/0–2	1.52 (0.53–4.35)	0.369
*TP53* mutation site	LSH L3/others	1.35 (0.47–3.90)	0.561
*IgH*/*MYC* translocation	Translocation/none	2.29 (0.73–7.14)	0.089

LSH, loop-sheet-helix; L3, Loop 3.

## Data Availability

Data are available from the corresponding author upon reasonable request.

## Supplementary Materials

The following are available online at https://www.mdpi.com/article/10.3390/diagnostics11050844/s1, Figure S1. ROC evaluation of the performance of *TP53* MAF in predicting disease progression. Figure S2. Four patients (3, 7, 8, 9) who had a positive result with ctDNA level earlier than that with CT imaging. Table S1. Clinical characteristics of all the patients. Table S2. False positive rate and PROVEAN score. Table S3. Response to treatment in different groups of ctDNA profile.

## Abbreviations

CAR T	Chimeric antigen receptor T
NHL	Non-Hodgkin’s lymphoma
MRD	Minimal residual disease
ctDNA	Circulating tumor DNA
ddPCR	Droplet digital PCR
OS	Overall survival
PFS	Progression-free survival
PB	Peripheral blood
MAF	Mutant allele fraction
PROVEAN	Protein Variation Effect Analyzer
NGS	Next-generation sequencing
IWG	International Working Group
LSH	Loop-sheet-helix
HR	Hazard ratio
DLBCL	Diffuse large B cell lymphoma
FL	Follicular lymphoma
AUC	Area under the curve
ROC	Receiver operating characteristic
