# Supplementary material for: TP53-Mutated Circulating Tumor DNA for Disease Monitoring in Lymphoma Patients after CAR T Cell Therapy
Source: Diagnostics (Basel). 2021 May 8;11(5):844. doi: 10.3390/diagnostics11050844 (PMC8151854; doi:10.3390/diagnostics11050844)
Supplement: Supplementary file 1 [file diagnostics-11-00844-s001.zip › diagnostics-1180379-supplementary.pdf]

Supplemental files

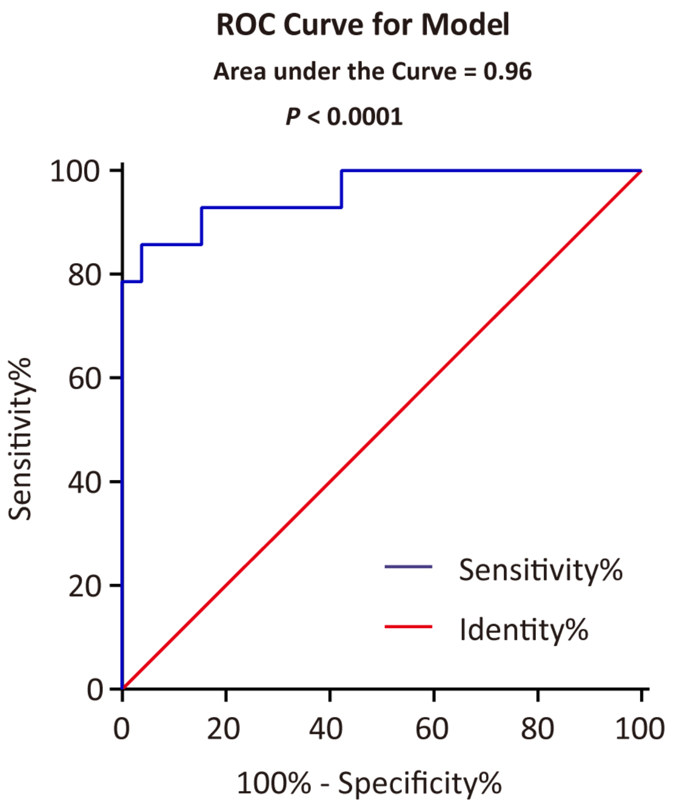

Figure S1. ROC evaluation of the performance of TP53 MAF in predicting disease progression.

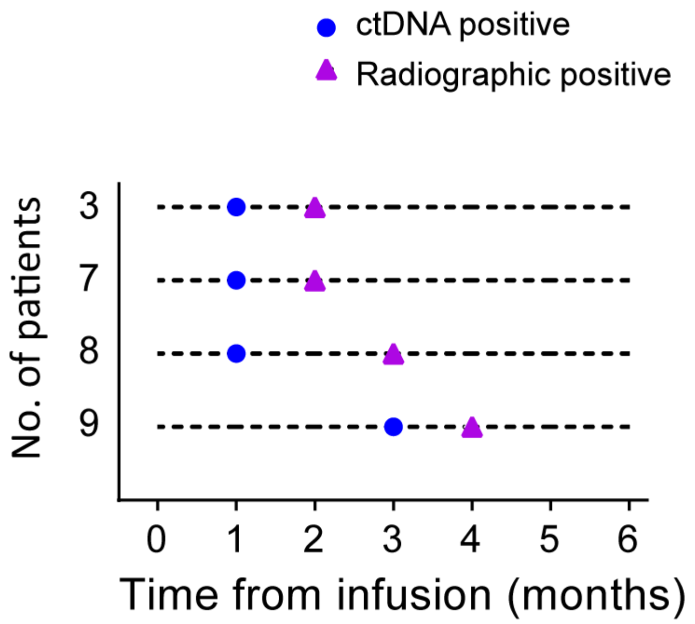

Figure S2. Four patients (3, 7, 8,9) that who has positive result in ctDNA level earlier than that with CT imaging.

Table S1. Clinical characteristics of all the patients.

No. of Patient	Gender	Age	Ann-Arbor Stage	IPI Score	Volumn > 5cm	Disease Status	Diagnosis	Nucleotide Change	Mutation Site	with 17p Loss	IgH/MYC Translocation
1	M	45	Ⅳ	3	No	Primary refractory	DLBCL	c.437G>A	outside	TP53 mutation only	No
2	M	64	Ⅳ	3	Yes	≥ Second relapse	DLBCL	c.818G>A	LSH	TP53 mutation only	No
3	M	38	Ⅳ	4	Yes	≥ Second relapse	FL	c.376T>A	LSH	TP53 mutation only	Yes
4	M	29	Ⅲ	4	Yes	First relapse	FL	c.818G>A	LSH	TP53 mutation only	Yes
5	F	34	Ⅳ	1	No	≥ Second relapse	DLBCL	c.695T>A	outside	TP53 mutation only	No
6	M	27	Ⅱ	4	No	Primary refractory	Burkitt	c.376-1G>A	LSH	TP53 mutation only	Yes
7	M	43	Ⅳ	3	Yes	First relapse	FL	c.733G>A	L3	TP53 mutation only	No
8	F	33	Ⅳ	5	Yes	Primary refractory	DLBCL	c.743G>A	L3	TP53 mutation only	No
9	M	52	Ⅳ	2	No	First relapse	DLBCL	c.714T-G	L3	with del (17p)	Yes
10	M	56	Ⅱ	2	Yes	Primary refractory	DLBCL	c.799C>T	outside	TP53 mutation only	Yes
11	M	43	Ⅳ	3	Yes	Primary refractory	DLBCL	c.725G>A	L3	TP53 mutation only	No
12	M	47	Ⅲ	2	Yes	≥ Second relapse	DLBCL	c.847-848insATCGGG	LSH	TP53 mutation only	No
13	M	47	Ⅳ	3	No	Primary refractory	DLBCL	c.733G>A	L3	TP53 mutation only	No
14	M	54	Ⅳ	3	Yes	Primary refractory	MCL	c.715A>G	L3	TP53 mutation only	No
15	M	49	Ⅳ	3	No	Primary refractory	MCL	c.488A>G	outside	TP53 mutation only	No
16	M	41	Ⅳ	1	No	First relapse	DLBCL	c.842A>T	LSH	TP53 mutation only	No
17	F	37	Ⅲ	1	No	Primary refractory	DLBCL	c.817C>T	LSH	with del (17p)	Yes
18	M	39	Ⅳ	4	Yes	≥ Second relapse	DLBCL	c.743G>A	L3	TP53 mutation only	No
19	M	37	Ⅳ	3	No	First relapse	DLBCL	c.622G>T	outside	TP53 mutation only	Yes
20	M	40	Ⅱ	3	No	Primary refractory	HGBL	c.524G>A	L2	TP53 mutation only	Yes
21	M	54	Ⅳ	1	No	Primary refractory	DLBCL	c.743G>A	L3	with del (17p)	Yes
22	M	40	Ⅳ	1	No	First relapse	MCL	c.756delC	outside	with del (17p)	Yes
23	F	56	Ⅲ	3	Yes	Primary refractory	DLBCL	c.524G>A	L2	TP53 mutation only	No
24	M	44	Ⅲ	3	No	First relapse	DLBCL	c.413C>T	outside	TP53 mutation only	No
25	F	60	Ⅳ	3	Yes	Primary refractory	DLBCL	c.637C>T	outside	TP53 mutation only	No
26	M	58	Ⅳ	1	Yes	Primary refractory	DLBCL	c.638G>A	outside	TP53 mutation only	No
27	M	29	Ⅲ	1	Yes	Primary refractory	DLBCL	c.743G>A	L3	TP53 mutation only	No
28	M	42	Ⅲ	3	No	First relapse	DLBCL	c.1007_1020del	outside	TP53 mutation only	No
29	M	42	Ⅳ	2	No	Primary refractory	DLBCL	c.844C>T	LSH	with del (17p)	No
30	M	32	Ⅳ	3	No	Primary refractory	DLBCL	c.700T>G	outside	TP53 mutation only	No
31	M	53	Ⅲ	2	No	First relapse	DLBCL	c.733G>A	L3	TP53 mutation only	No
32	F	38	Ⅳ	3	No	Primary refractory	DLBCL	c.437G>A	outside	TP53 mutation only	No
33	M	39	Ⅳ	3	Yes	First relapse	DLBCL	c.706T>G	outside	TP53 mutation only	Yes
34	F	43	Ⅳ	3	Yes	Primary refractory	DLBCL	c.818G>A	LSH	TP53 mutation only	No
35	M	55	Ⅳ	2	No	≥ Second relapse	DLBCL	c.742C>T	L3	TP53 mutation only	No
36	M	52	Ⅰ	1	Yes	≥ Second relapse	DLBCL	c.715A>G	L3	TP53 mutation only	No
37	M	49	Ⅳ	2	No	First relapse	DLBCL	c.744A>C	outside	TP53 mutation only	Yes
38	F	50	Ⅳ	3	No	≥ Second relapse	DLBCL	c.818G>A	LSH	with del (17p)	Yes
39	M	51	Ⅳ	2	Yes	First relapse	DLBCL	c.747G>C	L3	with del (17p)	No
40	M	56	Ⅰ	1	No	≥ Second relapse	PCNSL	c.318C>G	outside	TP53 mutation only	No

Table S2. False positive rate and PROVEAN score.

No. of Patient	Protein Change	Nucleotide Change	False positive Rate	Threshold	COSMIC ID	PROVEAN Score	Prediction (Cutoff = −2.5)
28	p.E336fs	c.1007_1020del	0.0001	0.0002	not available	/	/
40	p.S106R	c.318C>G	0.0001	0.0003	45944	−1.171	Neutral
6	-	c.376-1G>A	0.0000	0.0000	6900	/	/
3	p.Y126N	c.376T>A	0.0000	0.0000	44380	−8.317	Deleterious
24	p.A138V	c.413C>T	0.0005	0.0014	43818	−3.848	Deleterious
1 32	p.W146*	c.437G>A	0.0000	0.0001	43609	/	/
15	p.Y163C	c.488A>G	0.0000	0.0001	10808	−8.623	Deleterious
20 23	p.R175H	c.524G>A	0.0001	0.0002	43629	−4.872	Deleterious
19	p.D208Y	c.622G>T	0.0001	0.0003	10726	−7.998	Deleterious
25	p.R213*	c.637C>T	0.0001	0.0002	10654	/	/
26	p.R213Q	c.638G>A	0.0001	0.0003	10735	−3.753	Deleterious
5	p.I232N	c.695T>A	0.0000	0.0000	not available	−6.312	Deleterious
30	p.Y234D	c.700T>G	0.0000	0.0000	43768	−7.736	Deleterious
33	p.Y236D	c.706T>G	0.0000	0.0001	43602	−7.14	Deleterious
9	p.C238W	c.714T>G	0.0001	0.0003	44676	−10.733	Deleterious
14 36	p.N239D	c.715A>G	0.0005	0.0014	10777	−4.54	Deleterious
11	p.C242Y	c.725G>A	0.0000	0.0000	43713	−10.742	Deleterious
7 13 31	p.G245S	c.733G>A	0.0005	0.0015	10659	−5.877	Deleterious
35	p.R248W	c.742C>T	0.0000	0.0001	10656	−7.826	Deleterious
8 18 21 27	p.R248Q	c.743G>A	0.0001	0.0002	10662	−3.915	Deleterious
39	p.R249S	c.747G>C	0.0000	0.0001	10785	−5.864	Deleterious
22	p.T253Pfs*92	c.756delC	0.0003	0.0007	44623	−7.659	Deleterious
37	E258D	c.774A>C	0.0000	0.0001	46410	−2.911	Deleterious
10	p.R267W	c.799C>T	0.0001	0.0002	11183	−7.651	Deleterious
17	p.R273C	c.817C>T	0.0000	0.0000	48817	−4.775	Deleterious
2 4 34 38	p.R273H	c.818G>A	0.0002	0.0005	10660	−8.614	Deleterious
16	p.D281V	c.842A>T	0.0001	0.0003	11152	−7.65	Deleterious
29	p.R282W	c.844C>T	0.0000	0.0000	10704	−10.263	Deleterious
12	p.R282-R283insHR	c.847-848insATCGGG	0.0003	0.0005	not available	/	/

Score thresholds for prediction is −2.5, that is: Variants with a score equal to or below −2.5 are considered "deleterious,"; Variants with a score above −2.5 are considered "neutral".

Table S3. Response to treatment in different group of ctDNA profile.

Variable	Favorable	Unfavorable
Response—no. (%)		
Complete response (CR)	20	0
Partial response (PR)	5	1
Stable disease (SD)	0	2
Progressive disease (PD)	2	10
Objective response rate (CR + PR)% of patients	92.6%	7.7%
